# Identification of the Biomechanical Response of the Muscles That Contract the Most during Disfluencies in Stuttered Speech

**DOI:** 10.3390/s24082629

**Published:** 2024-04-20

**Authors:** Edu Marin, Nicole Unsihuay, Victoria E. Abarca, Dante A. Elias

**Affiliations:** Biomechanics and Applied Robotics Research Laboratory, Pontificia Universidad Católica del Perú, Lima 15088, Peru; edu.marin@pucp.edu.pe (E.M.); nicole.unsihuay@pucp.edu.pe (N.U.); delias@pucp.pe (D.A.E.)

**Keywords:** stuttering, speech language pathology, fluency disorders, EMG

## Abstract

Stuttering, affecting approximately 1% of the global population, is a complex speech disorder significantly impacting individuals’ quality of life. Prior studies using electromyography (EMG) to examine orofacial muscle activity in stuttering have presented mixed results, highlighting the variability in neuromuscular responses during stuttering episodes. Fifty-five participants with stuttering and 30 individuals without stuttering, aged between 18 and 40, participated in the study. EMG signals from five facial and cervical muscles were recorded during speech tasks and analyzed for mean amplitude and frequency activity in the 5–15 Hz range to identify significant differences. Upon analysis of the 5–15 Hz frequency range, a higher average amplitude was observed in the zygomaticus major muscle for participants while stuttering (*p* < 0.05). Additionally, when assessing the overall EMG signal amplitude, a higher average amplitude was observed in samples obtained from disfluencies in participants who did not stutter, particularly in the depressor anguli oris muscle (*p* < 0.05). Significant differences in muscle activity were observed between the two groups, particularly in the depressor anguli oris and zygomaticus major muscles. These results suggest that the underlying neuromuscular mechanisms of stuttering might involve subtle aspects of timing and coordination in muscle activation. Therefore, these findings may contribute to the field of biosensors by providing valuable perspectives on neuromuscular mechanisms and the relevance of electromyography in stuttering research. Further research in this area has the potential to advance the development of biosensor technology for language-related applications and therapeutic interventions in stuttering.

## 1. Introduction

Stuttering is a speech disorder that significantly affects the quality of life and communication abilities of people who experience it. Fluent speech depends on the harmonious interaction of the jaw, lips, throat, larynx, and middle ear muscles. However, this coordination is disrupted in stutterers, leading to abnormal muscle tremors and increased activity, especially before speech difficulties are noticed [1]. Studies have shown that stuttering extends beyond the mere disruption of speech fluency, deeply affecting individuals’ psychological well-being. For instance, it was found that adults who stutter are at a greater risk of developing chronic anxiety compared to non-stuttering individuals [2,3]. In addition, significant differences were found between adults who stutter and a control group on quality of life dimensions such as general health, emotional health, and social function [4].

Although it affects approximately 1% of the world’s population [5], its exact origins still need to be fully understood [6]. Many researchers believe that the development of stuttering may involve central nervous system function and genetic and language learning factors [7]. Therefore, researching the physiological mechanisms and neurological processes underlying stuttering can provide valuable insights into developing more effective treatment strategies and enhance our understanding of this condition.

EMG is a versatile technique with applications in various fields, including medical research, rehabilitation, ergonomics, and exercise science. In speech analysis, researchers have utilized EMG to explore different aspects, such as speech-motor control, muscle function assessment during speech therapy, and occupational voice use [8,9].

Regarding stuttering, EMG has proven invaluable in analyzing muscle activity during speech and characterizing the associated contractions. Numerous studies have focused on analyzing EMG signals, specifically from facial and neck muscles, primarily in adults who stutter [10,11]. These investigations typically involve the placement of surface electrodes on the skin to capture EMG signals, enabling researchers to assess parameters like muscle activity amplitude and duration.

Our extensive review of the literature from databases such as PubMed and IEEE Xplore from 1970 to 2023 highlights that, while the physiological mechanisms of stuttering, particularly regarding muscle activity during speech, have been explored, results remain inconclusive. Some of these studies have observed key trends and findings in the muscle activity of adults and children who stutter. A common assumption was the higher amplitude of muscle tension in people who stutter. Therefore, early studies have attempted to resolve the hypothesis that excessive or high-amplitude muscle activity occurs during disfluencies. These early studies, with a minimal number of adults, supported this claim [12,13]. However, in subsequent studies with a larger number of adults with stuttering, no relationship was found between higher electromyography (EMG) amplitudes and the orofacial, mandibular, laryngeal, and respiratory muscles during stuttering [14,15,16]. In addition, lateralization and, thus, asymmetry of muscle activity in the muscles of the face [17,18] and involuntary oscillations of muscle activity in the 5–15 Hz frequency band have been observed in some adults who stutter [14,19]. However, in children who stutter, the same oscillations in the 5–15 Hz band have not been found, and the presence of tremors was low, concluding that there is no significant relationship between stuttering episodes and muscle activity [20]. The outcomes of these studies have yet to provide explicit or definitive conclusions regarding the electrical muscle activity of speech muscles and their relation to stuttering due to the discrepancies among the results obtained. As previously mentioned, while some studies demonstrate a clear relationship, others do not. It was also found that studies similar to the current research, which focuses on the electrical muscular activity of speech muscles during stuttering, were carried out until 2013. Notably, the most recent study within this definition was conducted on preschool children by Walsh and Smith [20].

Recent trends in stuttering research have shifted towards the integration of electroencephalography (EEG) and advanced machine learning techniques, moving away from traditional electromyography (EMG) methods. This shift is highlighted by innovative studies such as the development of “TranStutter”, a deep learning model that significantly improves the classification of stuttered speech through 2D Mel-Spectrogram visualization and attention-based feature representation, achieving impressive accuracies on diverse datasets [21]. Additionally, EEG-based research has provided new insights into the neural dynamics of stuttering, revealing how the severity of stuttering correlates with changes in brain activity during speech preparation [22,23]. These advancements underscore a broader, more nuanced understanding of stuttering, emphasizing the importance of exploring neural mechanisms and leveraging technological innovations for diagnosis and treatment.

To understand and treat stuttering, there is still much to be discovered about its underlying mechanisms. In this regard, identifying the most active speech muscles during stuttering using EMG sensors is an essential step for optimizing the understanding of stuttering. This research can contribute additional information to previously found results and refine or lend more credence to established theories, such as the one postulating a direct relationship between the electrical activity of speech muscles and stuttering or the presence of higher activity at specific frequencies. Extending from these findings, the current research opens the possibility of developing biosensors specifically designed for stuttering events. These biosensors, informed by detailed EMG data, could lead to novel methods for monitoring and treatment of stuttering. For instance, the 2023 proposal “Speak in Public: an Innovative Tool for the Treatment of Stuttering through Virtual Reality, Biosensors, and Speech Emotion Recognition” demonstrates the practical application of biosensors to track a patient’s biological progress during treatment. Comprehensive data, including body temperature, heart rate, and electrodermal activity (EDA), can be collected using biosensors. Doctors can gain real-time access to the patient’s physiological responses by using biosensors during treatment. This valuable data allow therapists to identify specific stress triggers in people who stutter, making it easier to tailor treatment strategies to individual needs. The integration of biosensors not only improves the treatment process but paves the way for more personalized and practical support for patients undergoing stuttering treatment [24]. Therefore, this research deepens our understanding of stuttering at a physiological level and catalyzes the development of technological solutions for its management.

This study identifies the biomechanical responses of orofacial muscles during stuttering to uncover which muscles are most affected in terms of EMG activity during stuttering episodes. By comparing individuals with and without stuttering, we aim to identify distinct patterns of muscle activity associated with speech disfluencies. Fluent and disfluent speech was examined to determine how stuttering impacts muscle activity during various speech contexts. The study’s approach involves analyzing speech samples collected under different conditions and for different types of disfluencies to ensure a comprehensive understanding of the physiological aspects of stuttering.

## 2. Materials and Methods

### 2.1. Ethical Aspects

A document was prepared that provided a detailed description of the study, including the number of participants, the primary protocol, the inclusion and exclusion criteria, the procedures for accessing and administering the obtained data, the data analysis, the biosafety measures, and the informed consent process, which was submitted to the Research Ethics Committee for Life Sciences and Technologies of the Pontificia Universidad Católica del Perú. Upon its receipt on 15 July 2022, the Committee reviewed and approved it, assigning the reference number N° 004-2022-CEICVyTech/PUCP.

### 2.2. Inclusion Criteria for Participants

Group A consisted of adults between 18 and 40 who were not diagnosed with language and speech difficulties, including language development disorders, stuttering, tachyphemia, tachykalemia, or phonological disorders. Group B comprised adults between 18 and 40 who experienced stuttering episodes in their speech. Individuals with other speech difficulties and motor disabilities were excluded from this group.

### 2.3. Exclusion Criteria for Participants

Exclusion criteria were applied for participants in both groups, resulting in the exclusion of those participants who presented motor difficulties or disabilities, such as cerebral palsy, dyspraxia, motor apraxia, and other problems related to coordination, limited reach, reduced strength, unintelligible speech, and fine and gross motor difficulties. These criteria are meant to prevent these conditions from affecting the test performance and interpretation of the results. Additionally, individuals with known allergies to silver were excluded to avoid potential adverse reactions due to the silver electrodes used in the sensors.

### 2.4. Sample Size and Recruitment

The study considered the statistical variables of population, confidence level, and margin of error to determine the necessary sample size. According to previous studies [5], the prevalence of stuttering in the population is 1% (*p* = 0.01). Extrapolating this statistic to the Peruvian context, it was estimated that around 137,790 people between 18 and 40 years old experience stuttering, and a specific population of youth and young adults numbering 13,779,000 Peruvians living in Lima was considered [25].

In determining the necessary sample size, Equation (1) was utilized, suitable for an infinite population as per Camacho-Sandoval [26]. In this equation, $Z_{\alpha }$ denotes the confidence level, *p* represents the percentage prevalence of the population, and *d* signifies the maximum permissible error. This study used a confidence level of 95% ($Z_{\alpha }$ = 1.96) and a maximum permitted error of 3% (*d* = 0.03).

(1) $$n_{0}=\frac{Z_{\alpha }^{2}\times p\left(1-p\right)}{d^{2}}$$

These criteria ensure that the obtained sample adequately represents the population under study. The necessary calculations were performed, and it was determined that a minimum of 43 participants would be required. However, for this research, an optimal sample of 55 youth and young adults from Lima was selected, comprising 34 men and 21 women aged between 18 and 40 years. No matching criteria based on sex or age were employed, except for the requirement of legal adulthood and an upper age limit of 40 years. This precaution was taken to mitigate the risk, particularly amid the backdrop of the COVID-19 pandemic.

On the other hand, minimal variability was expected in the data collected from the control group, consisting of individuals without stuttering. Therefore, a sample size of 30 individuals without stuttering would be sufficient. The total sample of 85 participants was divided into two groups: Group A, which consisted of 30 participants without stuttering, and Group B, which consisted of 55 participants with stuttering.

Group A participants were contacted through social networks and close circles of the researchers. Those who met the inclusion and exclusion criteria to participate in the research were invited, and any doubts were addressed. On the other hand, Group B participants were reached out to through open social networks and virtual support group pages dedicated to stuttering. Additionally, announcements and videos were published on the laboratory’s official website (GIRAB-PUCP).

### 2.5. Muscles: Selection and Biomechanical Variables

The electrical activity of the following muscles was analyzed: orbicularis oris, zygomaticus major, depressor anguli oris, sternocleidomastoid, and masseter. The choice of muscles for this study was based on a literature review of measurement of the EMG signal in patients who stutter or undergo laryngectomy. This review identified some facial and neck muscles that have been consistently studied in relevant research and are closely related to speech production and swallowing [20,27,28,29,30,31]. The selection of the muscle to study considered factors such as its contribution to stuttering or its recurrence during procedures related to laryngeal surgeries, its superficial position, which facilitated the non-invasive placement of electrodes on them, and the size or appropriate anatomical location for the precise measurement of myoelectric activity, among other similar things. The muscles selected were those of the orofacial region, such as the orbicularis oris and the zygomaticus major, and those located in the neck, such as the sternocleidomastoid. Additionally, the jaw muscle (masseter) and facial muscle (depressor anguli oris) were included because these muscles play a role in modulating phonation. These muscles were selected based on their functional relevance to laryngectomy for stuttering and evidence from previous research that provides invaluable information for understanding the mechanisms underlying these disorders [12,13].

In our analysis, we focused on extracting specific biomechanical variables from the EMG data to characterize muscle activity during stuttering episodes. Key features included the mean amplitude, which measures the average electrical activity across the muscle during a defined period, reflecting the overall activation level of the muscle fibers involved. Mean amplitude has demonstrated its effectiveness in evaluating the biomechanical properties of muscle activity during various motor tasks, including speech production [32,33].

In addition, a frequency analysis was performed in the 5–15 Hz range to obtain the differences in muscle activity. Previous studies have highlighted the presence of abnormal spikes and tremor-like oscillations in the electromyography (EMG) activity of the orofacial, jaw, larynx, and neck brace muscles in the range of 5 to 15 Hz, which suggests a specific association between this frequency band and the manifestation of stuttering symptoms [14,15,16,19,34]. Higher amplitude oscillations in this region have been consistently associated with stuttering, suggesting a possible link between neuromuscular activity and the disorder [14,35,36]. These results highlight the extreme heterogeneity in muscle activation patterns underlying stuttering. Self-reports of stuttering indicate that these disorders are often accompanied by physical tension [37], which various external stressors can exacerbate.

### 2.6. Materials

- Eight Delsys EMG Trigno Avanti Sensors, which have a sampling frequency between 2148 Hz and 4296 Hz, a resolution of 16 bits, a battery life between 4–8 h, and 8 sensors (27 × 37 × 13 mm) for 8 different muscles or muscle groups.
- Panasonic HC-V520M video camera with 16 GB internal memory and 720p resolution.
- A 64-bit laptop with a 2.0 GHz processor, 2 GB of system memory, and 128 MB of graphics memory.
- Nexcare Transpore 3 M adhesive tape.
- Disinfectant and exfoliating wipes.
- Printed informed consent sheets for the participants to sign.
- EMGWorks Acquisition software version 4.8.0 for data collection and Delsys File Utility software (https://delsys.com/emgworks/) for file conversion.
- MATLAB R2023a software for data processing and analysis.

### 2.7. Experimental Setup and Preparations

To ensure the smooth execution of the study, appointments were scheduled with considerations for participant and equipment readiness. The experimental setup was completed with necessary precautions to maintain a safe and controlled environment for all participants.

**Figure 1:**
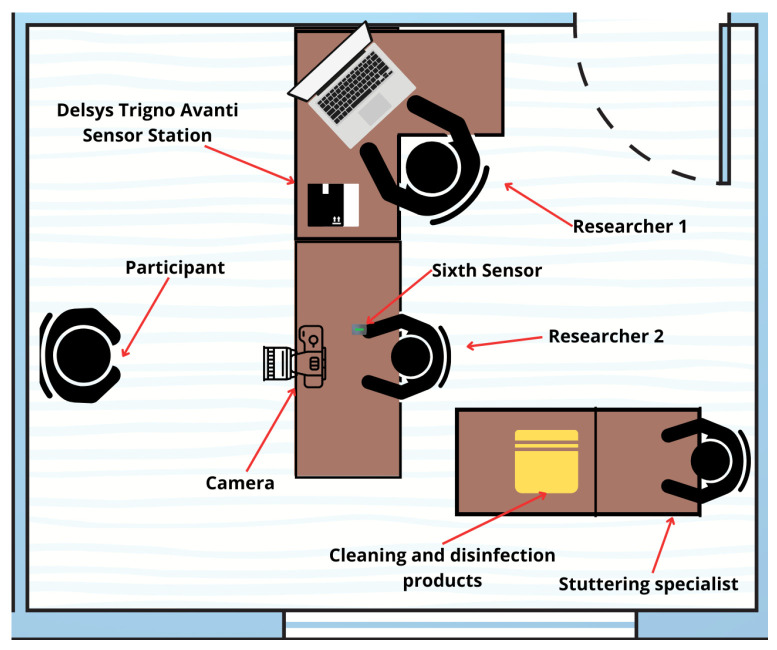
Graphic illustration of the work area used for the tests.

As shown in Figure 1, the experimental environment covered an area of 15 square meters and featured two desks. The first desk served as a placement for the sensors, laptop, and other accessories necessary for the experiment. The second desk was for placing the informed consent form, as well as disinfection and cleaning items for the use of the participants. The researchers positioned the camera on the first desk, ensuring it focused on the chair where they conducted participant interviews. They securely mounted it using a tripod or base if space was available.

### 2.8. During the Procedure

**Figure 2:**
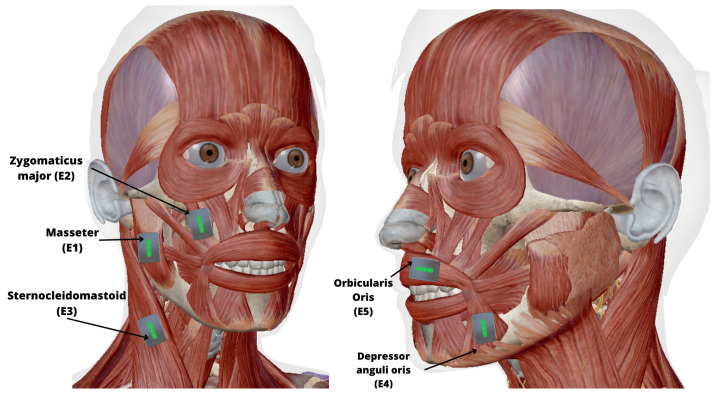
Graphic illustration of the distribution of the sensors around the face and neck: E1. Masseter, E2. Zygomaticus major, E3. Sternocleidomastoid, E4. Depressor anguli oris, E5. Orbicularis oris.

All participants, with or without stuttering, voluntarily consented to join the study, understanding their rights to privacy and withdrawal, with no financial incentives involved. The five Delsys EMG sensors were placed in the following muscles: masseter (E1); zygomaticus major (E2); sternocleidomastoid (E3); depressor anguli oris (E4); and orbicularis oris (E5), as seen in Figure 2. It should be noted that a sixth sensor was used as a control in Group B so that, when one of the researchers pressed it, a signal peak was recorded each time a stuttering episode was detected, helping with the subsequent synchronization of the signal and the video. In addition to the video recording, a protocol approved by the ethics committee was followed, where the voice was recorded exclusively to identify moments of disfluency after obtaining informed consent from the participants. Likewise, when placing the EMG sensors, it was verified that the reference arrow of the sensor was aligned with the direction of the fibers of the muscle to be analyzed. For better support and comfort of the participant, the contact between the electrodes and the skin was ensured by Nexcare Transpore 3M adhesive tape.

The electrodes were placed, and the sensors were synchronized with the EMG acquisition software, with a sampling frequency setting of 2148 Hz, a range of 11 mV, and a bandwidth of 20–450 Hz. After completing the software setup, the trial was initiated by turning on the chamber.

During the protocol, four tests were performed: the first consisted of a spontaneous speech evaluation in which the participant was asked questions about their personal data, medical history, and educational background. The second test was a read-aloud of a text provided to the participant. A stuttering specialist chose this text to be of appropriate length and adequate phonetic balance. The third test consisted of a telephone call to a trusted person to establish a casual conversation of approximately one minute. Finally, the fourth test focused on a brief presentation of roughly three minutes on any topic of interest to the participant. These tests were adapted from the Stuttering Severity Instrument - Fourth edition (SSI4) [38] by the stuttering specialist, who was present during the evaluation. The specialist also ensured that, despite sensors, speech was conducted as naturally as possible.

After completing the tests, the camera and software recording were stopped to remove the sensors from the participant’s face. Disinfectant wipes were provided for the participants’ faces, and the sensors were disinfected for later use.

### 2.9. Data Collection

Samples of fluent speech and stuttering were collected from Groups A and B. For each participant, 40 electromyography (EMG) signal samples with a duration of 1 s were acquired during fluent speech. Similarly, all samples with the same period were extracted during disfluent speech.

To ensure the fluency of the speech samples, those containing any speech with disfluencies were excluded, and the selection of adjacent samples with a minimum time interval of 0.5 s was avoided. On the other hand, speech samples with disfluencies were carefully selected so that these were contained partially or entirely within the time interval.

Disfluencies were classified with the help of the stuttering specialist in repetitions, prolongations, and blocks. Repetitions are characterized by repeating sounds or syllables within words, often indicative of an attempt to initiate speech flow. Prolongations involve the unnatural stretching of a sound beyond its typical duration, reflecting difficulty in speech continuation. Blocks represent moments when speech is halted or interrupted at the onset of a word or sound, signaling a significant disruption in speech production. These definitions were adopted to provide a solid basis for examining EMG responses in orofacial muscles to these specific stuttering behaviors.

### 2.10. Data Analysis

This study divided the collected data into two groups (A and B). For each participant in both groups, five EMG frames were acquired, corresponding to each muscle or muscle group, resulting in a total of 475 EMG frames to be processed. The “Delsys File Utility” tool was used to extract the EMG frames and convert them from “.hpf” to “.mat” format to perform the corresponding analysis. The EMG frames were between 650 and 1800 s long and operated on a millivolt (mV) scale.

Subsequently, two filters were designed; the first one consists of a low-pass FIR filter of order 20 with a cutoff frequency of 400 Hz to filter the EMG signal and eliminate the high-frequency noise. The second one consists of an FIR high-pass filter with a cutoff frequency of 20 Hz [39] to remove low frequencies. The last one is an FIR band-reject filter to eliminate the 60 Hz interference and its harmonics, thus avoiding ambient noise from the power grid.

The frequency spectrum of the filtered signal was calculated using the discrete Fourier transform (DFT), and the amplitude and power spectra were obtained. Then, the RMS envelope of the signal was found, and the rectified signal and the linear envelope were plotted. Finally, the RMS value of the signal was calculated and obtained.

First, the analysis was run per participant. After applying a 60 Hz noise filter to the signal, the amplitude of the EMG signal of the samples of fluent speech and speech with disfluencies was compared. For this purpose, the signal integral (IEMG) was performed, and the average was obtained for the speech and disfluency samples. In the case of frequency analysis, the power spectral density was calculated for each of the samples, and the signal integral was calculated in the 5–15 Hz range; in addition, the total signal integral was obtained to calculate the percentage of muscle activity in the 5–15 Hz range concerning the total. This analysis was performed for each muscle in a participant, and the data obtained were stored in the “.mat” format for subsequent analysis.

Once the muscle activity data were obtained for each muscle and each participant, we conducted a comprehensive evaluation of each muscle across all participants to identify significant differences in EMG activity between fluent speech and speech with disfluencies (*p* < 0.05). Data normalization was implemented using the mean activity normalization method to ensure rigorous analysis, which involved dividing the EMG data by the mean activity. This approach was critical for controlling inter-participant variability and mitigating the influence of any external factors on the EMG signal. Furthermore, the Shapiro–Wilk test was conducted to confirm the data’s adherence to normality assumptions required for the *t*-test. The statistical Student *t*-test from the MATLAB statistics and machine learning toolbox was utilized to identify muscles with statistically significant differences. This analytical framework was consistently applied across comparisons between Group A and Group B for both disfluent and fluent speech and Group A fluent speech versus Group B disfluencies.

Importantly, the analysis extended beyond simple comparisons between groups. We divided the data based on the context of speech acquisition into four distinct tests involving spontaneous speech evaluation, reading aloud, telephone conversation, and a brief presentation. This division allowed for an in-depth speech context analysis, providing a nuanced understanding of whether observed differences in muscle activity could be attributed to stuttering or vary due to the nature of the utterances in different tests. Additionally, categorizing speech samples based on specific disfluencies—repetitions, prolongations, and blocks—enabled a more detailed examination of stuttering manifestations across contexts. This was done to ensure that our findings robustly differentiate between the physiological markers of stuttering and the variability introduced by different speech tasks, thereby addressing potential biases.

## 3. Results

### 3.1. Comparison between Groups (A and B)

In both amplitude and frequency analysis, five specific muscles were evaluated: the depressor anguli oris (DAO), orbicularis oris (OO), masseter (M), sternocleidomastoid (S), and zygomaticus major (ZM).

**Figure 3:**
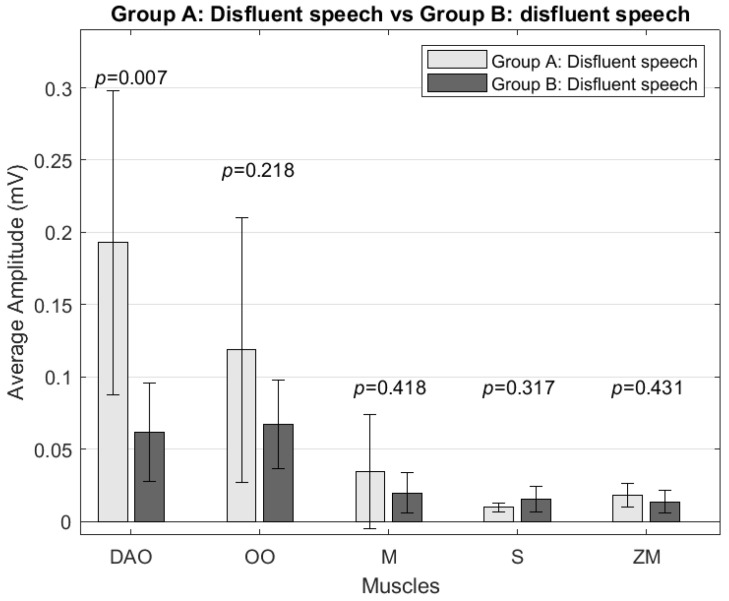
Comparison of average amplitude (mV) between Group A and Group B’s disfluent speech for the depressor anguli oris (DAO), orbicularis oris (OO), masseter (M), sternocleidomastoid (S), and zygomaticus major (ZM) muscles.

In the amplitude analysis, no significant differences were found in the signal amplitude for any muscle when comparing the fluent speech samples of both groups and the fluent speech samples of Group A with the speech samples with disfluencies from Group B. However, significant differences (*p* = 0.0071 and *p* = 0.0052, respectively) in the depressor anguli oris muscle amplitude were observed when comparing Group A’s disfluency speech samples with Group B’s disfluency speech and fluent speech samples. The depressor anguli oris muscle amplitude was higher in Group A by 214% compared to disfluent speech (Figure 3) and 236% compared to Group B’s fluent speech.

**Figure 4:**
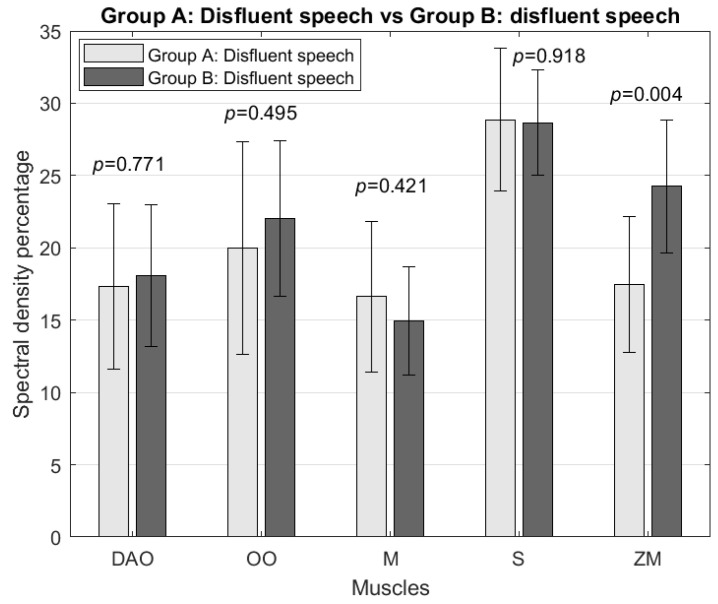
Comparison of spectral density percentage in the 5–15 Hz range between Group A’s and Group B’s disfluent speech for the depressor anguli oris (DAO), orbicularis oris (OO), masseter (M), sternocleidomastoid (S), and zygomaticus major (ZM) muscles.

As for the analysis of the activity in the frequency range 5–15 Hz, significant differences were found concerning the activity of the zygomaticus major muscle; this was higher in the samples of Group B disfluencies than in the samples of Group A fluent speech by 47% and that of disfluencies by 39% (Figure 4), corresponding to *p* = 0.0004 and *p* = 0.0041, respectively. Similarly, Group B’s fluent speech samples were more significant than Group A’s fluent speech samples by 51% and disfluencies by 43%, corresponding to *p* = 0.0001 and *p* = 0.0016, respectively.

### 3.2. Group A: Fluent Speech and Speech with Disfluencies

**Figure 5:**
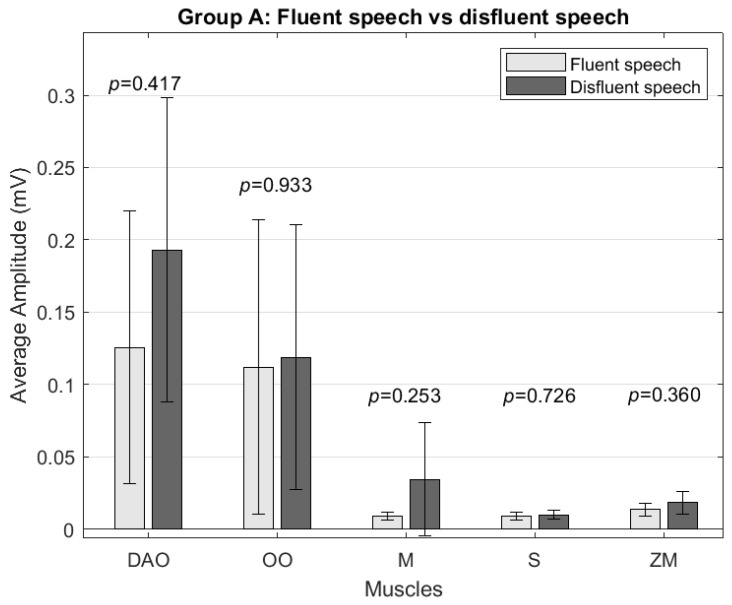
Comparison of average amplitude (mV) between Group A’s disfluent and fluent speech for the depressor anguli oris (DAO), orbicularis oris (OO), masseter (M), sternocleidomastoid (S), and zygomaticus major (ZM) muscles.

In Group A, a slightly higher average amplitude was found for the samples with disfluencies. However, no significant differences were found between fluent speech and speech with disfluencies (Figure 5). In the analysis of muscle activity at a frequency of 5–15 Hz, no significant differences were found in any muscle.

### 3.3. Group B: Fluent Speech and Speech with Disfluencies

**Figure 6:**
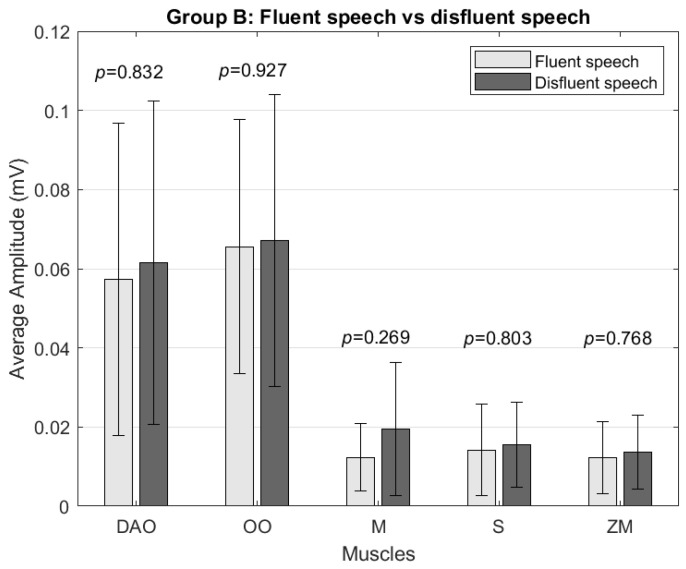
Comparison of average amplitude (mV) between Group B’s disfluent and fluent speech for the depressor anguli oris (DAO), orbicularis oris (OO), masseter (M), sternocleidomastoid (S), and zygomaticus major (ZM) muscles.

In Group B, no significant differences were found between the average signal amplitude of fluent speech or speech with disfluencies. For all muscles, a slightly greater amplitude was presented in the case of speech with disfluencies (Figure 6). In the analysis of muscle activity at a frequency of 5–15 Hz, no significant differences were found in any muscle.

### 3.4. Analysis in Context

When the same analysis was performed, isolating the different phases of the test (personal data, reading, call, and exposition), slight variation was found in the results. The average amplitude calculated was broadly similar; however, in the case of the depressor anguli oris, the disfluency samples of Group A presented a higher amplitude (*p* = 0.0208) than the disfluencies of Group B by 208% for samples collected during calls. Additionally, in the analysis of frequencies in the range of 5–15 Hz, in the case of speech in reading, it was found that when comparing the fluent speech of Group A with the fluent speech and disfluencies of Group B, the depressor anguli oris and masseter muscles presented slightly greater muscle activity concerning the general analysis.

**Figure 7:**
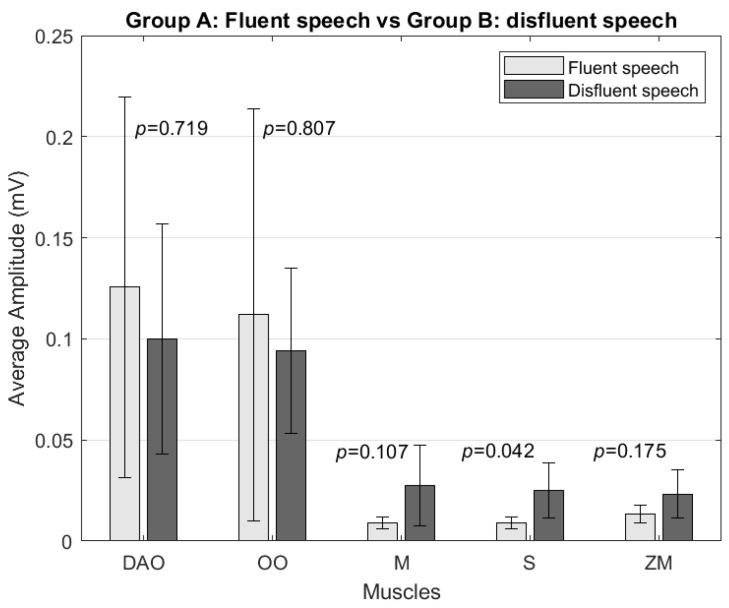
Comparison of average amplitude (mV) between Group A’s fluent speech and Group B’s disfluent speech during stuttering blocks for the depressor anguli oris (DAO), orbicularis oris (OO), masseter (M), sternocleidomastoid (S), and zygomaticus major (ZM) muscles.

Regarding the analysis of types of stuttering (repetitions, prolongations, blocks), it was found that, for repetitions, in the analysis of average amplitude, the zygomaticus major muscle had a significant difference (*p* = 0.0311), in which the disfluencies of Group A presented greater amplitude than those of Group B by 109%. In the case of the analysis of stuttering in prolongations, no differences were found in the patterns for the general analysis. In contrast, in the study of stuttering blocks, a higher average amplitude was observed for Group B disfluencies compared to Group A fluent speech (*p* = 0.0422) in the sternocleidomastoid muscle, as speech with disfluencies presented a higher amplitude by 180% (Figure 7). Finally, the analysis at frequencies 5–15 Hz for blocks was similar and consistent with the overall analysis.

## 4. Discussion

This study evaluated activation patterns of five facial and neck muscles in adults who do not stutter (Group A) and adults who stutter (Group B). The amplitude and activity analysis results in the 5–15 Hz frequency band were compared.

### 4.1. Comparison between Groups (A and B)

In this study, significant differences in the amplitude of the depressor anguli oris muscle amplitude were observed when comparing disfluent speech samples from Group A with fluent and disfluent speech samples from Group B. These findings may contradict previous findings [8,9] and indicate that, during stuttering, some muscles may have less overall muscle activation, such as the depressor anguli oris in adult stutterers.

Regarding the analysis at frequencies in the 5–15 Hz range, significant differences were observed in the activity of the zygomaticus major muscle. The activity was higher in Group B compared to Group A in all measurements, even between Group B’s fluent speech samples and Group A’s disfluent speech samples. This result is consistent with several previous studies [10,15], where it was stated that there is more significant activity in the jaw, lip, and laryngeal muscles in that frequency band; however, unlike previous studies, the statistically significant difference was found only in the zygomaticus major muscle, suggesting the unique role of orofacial muscles in speech fluency. This observation highlights the possible influence of emotional states and orofacial muscle tension on speech production, especially in individuals who stutter. Although the zygomaticus major muscle is primarily responsible for facial expressions [40], its increased activity during stuttering episodes may be due to increased emotional arousal or stress, indirectly affecting speech fluency, while the other muscles had more substantial activity but minimal differences.

The increased muscle activity in the 5–15 Hz frequency range for Group B compared to Group A suggests that the underlying neuromuscular mechanisms of stuttering may involve subtle aspects of synchronization and coordination in muscle activation crucial for the complex coordination required in the production of fluent speech. The increased activity within this specific frequency band could be associated with the fine-tuned neuromotor control required during speech, which could be altered in stuttering. This observation may point to more subtle aspects of neuromuscular dysfunction in stuttering, potentially related to the timing and coordination of muscle activation rather than the intensity of muscle use.

### 4.2. Intragroup Comparison: Fluent and Disfluent Speech

In Group A, no significant differences in EMG signal amplitude were found between fluent and disfluent speech samples. This finding is consistent with previous studies on disfluencies in adults who do not stutter [10,11,12].

On the other hand, in Group B, no significant differences in EMG signal amplitude between fluent and disfluent speech samples were found. These results are consistent with previous research that found no significant differences within the same study group [12]. Additionally, the results contradict the hypothesis that adults who stutter have more significant muscle activity in the facial muscles tested during stuttering episodes than in fluent speech.

Similarly, analysis at frequencies 5–15 Hz found no significant differences in comparing samples from the same fluent speech group and with disfluencies for either group. As with the amplitude analysis, the results show that, generally, in the same person, there is no difference in muscle activity between the disfluency samples and fluent speech in the 5–15 Hz frequency range.

### 4.3. Analysis of Stuttering in Context

The frequency of stuttering episodes varied between individuals and circumstances. Participants reported experiencing more blocking and stuttering when calling acquaintances, possibly due to the stress generated by the situation and the associated head movement. Occasionally, telephone calls can cause stressful situations that produce signal disturbances.

A lower frequency of stuttering episodes was observed in participants in comfortable situations compared to more stressful ones. In addition, increased participant stress levels were observed during speech pauses, resulting in a higher frequency and intensity of such pauses. These findings suggest that stress may play a crucial role in the occurrence and frequency of stuttering episodes. That context may also influence the manifestation of these episodes in participants who stutter.

The results of the context signal analysis indicate a higher average amplitude in the zygomaticus major muscle compared to the general analysis during phone calls, for samples with disfluencies in Group A compared to Group B, which could be related to stress or nervousness in the conversation. Subsequently, in the analysis of muscle activity in different types of disfluencies in stuttering, it is noteworthy that a higher average amplitude was found in the EMG signal of the sternocleidomastoid muscle during blocks, presenting a significant difference compared to Group A. The increased activity of the sternocleidomastoid muscle during blocks could suggest a link between neck muscle tension and the physical manifestation of stuttering. Considering that stuttering is a multifaceted disorder with sensory-motor, cognitive, and emotional dimensions, the EMG patterns observed may reflect the complex interaction between these components.

This approach enabled us to discern whether the observed variations in EMG responses were inherently linked to the stuttering phenomenon or were influenced by the distinct speech contexts of the tests. By analyzing speech in context, we could systematically evaluate the consistency of stuttering manifestations across varying communicative demands, thereby reinforcing the integrity of our findings. As we discussed, this study’s results tend to be similar on most muscles analyzed regardless of the speech context. Still, it is important to note that the differences found in the zygomaticus major muscle during samples from the phone calls were found for all types of samples containing disfluencies for both groups. This implies that, while the test could introduce bias in comparing fluent and disfluent speech, it does not affect the results when comparing the disfluent speech of both groups.

## 5. Conclusions

In summary, significant differences were observed in the mean depressor anguli oris muscle amplitude of Group A samples with disfluencies compared to Group B samples. Group B had lower mean depressor anguli oris muscle amplitude than Group A in this comparison. On the other hand, within the analysis of muscle activity in the 5–15 Hz frequency range, the zygomaticus major muscle presented significant differences, as the muscle activity was higher in Group B samples than in Group A. These findings imply that differences in muscle activity among individuals with stuttering may be more related to the frequency and neuromotor activation patterns, particularly in the 5–15 Hz range, rather than the amplitude of muscle use. This suggests that the underlying neuromuscular mechanisms of stuttering involve subtle aspects of timing and coordination in muscle activation crucial for the complex coordination required in fluent speech production.

Most importantly, the implications of these findings extend beyond the academic world. The study demonstrates promising applications in developing biosensor technology for speech-related interventions. By accurately identifying the most active speech muscles during stuttering and characterizing the neuromuscular activation patterns through EMG analysis, it becomes feasible to design biosensors that can detect and quantify these specific muscle activities. The development of these biosensors can be envisioned in two significant ways: first, as diagnostic tools that aid speech therapists and clinicians in assessing the severity and patterns of stuttering in individuals; second, as therapeutic devices that can be integrated into treatment protocols. Furthermore, integrating machine learning algorithms with EMG data from these biosensors can lead to personalized treatment and monitoring device development. In addition, the continuous data collection from these biosensors can contribute to a more comprehensive understanding of stuttering. The amassed data can be used for longitudinal studies, providing insights into the progression of stuttering over time and the effectiveness of various treatment strategies. Essentially, this research enriches the field of speech-language pathology, provides valuable insights, and paves the way for more effective and personalized approaches to supporting individuals who stutter.

Acknowledging the limitations of this study is crucial for a comprehensive understanding of its context and implications. Firstly, while sufficient for preliminary investigation, the sample size may only partially represent the diversity of stuttering severity and patterns across the broader population. Despite efforts to simulate natural speaking conditions, the laboratory environment can only partially replicate the variability and stressors in everyday communication that might influence stuttering behavior and muscle activity. Additionally, the use of Trigno™ sensors, although state-of-the-art for EMG data collection, introduces the challenge of sensor placement accuracy and the potential for discomfort that could affect speech production. These limitations underscore the need for larger-scale studies in more naturalistic settings and the exploration of additional physiological measurements to enrich our understanding of stuttering’s complex mechanisms.

## Data Availability

The data are available by contacting Dante A. Elias (girab@pucp.pe).

## Abbreviations

The following abbreviations are used in this manuscript:

PUCP	Pontificia Universidad Católica del Perú
EMG	Electromyography
IEMG	Integrated electromyography
FIR	Finite impulse response
DFT	Discrete Fourier transform
RMS	Root mean square
DAO	Depressor anguli oris
OO	Orbicularis oris
M	Masseter
S	Sternocleidomastoid
ZM	Zygomaticus major
